# Physical Activity and Mental Health Among Physicians in Tertiary Psychiatric Hospitals: A National Crosssectional Survey in China

**DOI:** 10.3389/fpsyg.2021.731525

**Published:** 2021-10-15

**Authors:** Jin Luo, Huanzhong Liu, Yuanli Liu, Feng Jiang, Yi-Lang Tang

**Affiliations:** ^1^School of International and Public Affairs, Shanghai Jiao Tong University, Shanghai, China; ^2^Department of Psychiatry, Chaohu Hospital of Anhui Medical University, Hefei, China; ^3^School of Health Policy and Management, Chinese Academy of Medical Sciences and Peking Union Medical College, Beijing, China; ^4^Department of Psychiatry and Behavioral Sciences, Emory University, Atlanta, GA, United States; ^5^Substance Abuse Treatment Program, Atlanta VA Medical Center, Decatur, GA, United States

**Keywords:** physical activity, happiness, physician, psychiatric hospital, China

## Abstract

This study aimed to examine the level of mental health and its correlates, particularly physical activity (PA) frequency, among physicians in tertiary psychiatric hospitals. In a national crosssectional survey, 4,520 physicians nested in 41 tertiary psychiatric hospitals from 29 provinces completed the online questionnaire. Their mean age was 38.5 ± 8.6 years, and 41.86% of physicians were men. More than one-third (35.24%) of physicians reported no PA in the past month, and only 21.88% reported happiness. Only 55.15 and 58.10% of the physicians reported normal status of depression and anxiety, respectively. In the adjusted multivariable ordinal logistic regression, higher PA frequency was associated with depression, anxiety, and happiness, except those who reported PA almost every day. Programs that aim to increase PA may promote the mental health of physicians in tertiary psychiatric hospitals.

## Introduction

Mental health can be defined as more than an absence or lack of mental disorders (Kumar et al., 2021). It is generally related to physical, social, and spiritual wellness, including positive moods, such as happiness. Happiness, an essential measure of overall well-being, is defined as the subjective state of mind characterized by enjoyment and contentment (Zhang et al., 2020). Happiness among physicians is an important research topic in recent decades (Werdecker and Esch, 2021). Many studies showed that a low level of happiness (unhappiness) not only affects the personal life and subjective well-being of physicians, but also impacts the healthcare quality that occurs due to medical errors or inefficacy (Wallace et al., 2009; Hall et al., 2016; Dewa et al., 2017). To date, few studies have focused on happiness among Chinese physicians.

Physical activity (PA), for example, physical exercise, sports, and physically active hobbies (Gottschlich et al., 2019), is known as an essential factor related to psychological health (Zhang and Chen, 2019). Several studies have documented a positive relationship between PA and happiness (Zhang and Chen, 2019). For example, in the Student Activity and Sports Participation Survey Ireland program, students with moderate and high physical activity according to the International Physical Activity Questionnaire-Short Form were more likely to be happy (Murphy et al., 2018). Based on the 2014 China Family Panel Study, residents with frequent PA (≥4 times/month) were more likely to have a higher subjective well-being (Xu et al., 2019). In the Study on Global Ageing and Adult Health (SAGE), the authors demonstrated that meeting physical activity guidelines (≥150 min of moderate-to-vigorous physical activity/week) was significantly associated with more happiness (Felez-Nobrega et al., 2021). Meanwhile, PA has also been proven as a promising adjunct intervention for mood disorders, including bipolar and major depressive disorder (Wang and Ashokan, 2021).

Mental health is an essential concern among Chinese physicians due to long working hours/day and heavy workload. According to a national survey, Chinese physicians generally worked 9.62 h/day, and 62.58% complained about hefty workload. Meanwhile, 31.28% of physicians reported burnout (Wu et al., 2019, 2020). In another survey, the prevalence of depressive symptoms in Chinese physicians was 42.3%, which was much higher than Chinese residents (Huang et al., 2019; Fu et al., 2021). Furthermore, a recent study found that nearly half of Chinese physicians working in intensive care units experienced psychological symptoms (Chen et al., 2021).

Physical activity is an essential factor for mental health, but few studies have examined the relationship between PA frequency and mental health among physicians (Zhang and Chen, 2019). To address this research gap, our national psychiatric hospital's survey provided a unique opportunity. We aimed to examine the association between PA frequency and mental health using this dataset, controlling for a series of covariates among Chinese physicians.

## Materials and Methods

### Study Design and Participants

This national crosssectional study was conducted between March 18 and 31, 2019. The National Health Commission of China approved this project. Totally 41 tertiary psychiatric hospitals were selected from 29 provinces, except Gansu and Tibet, due to the lack of tertiary psychiatric hospitals. All physicians in these target hospitals were recruited to participate in this survey. Each physician voluntarily responded to a smartphone-based questionnaire anonymously (Xia et al., 2020). The QR code for the survey was posted online and was distributed to potential participants *via* WeChat. On average, it took the participants 10–15 min to complete the questionnaire.

The Ethics Committee in Chaohu Hospital of Anhui Medical University approved the study protocol. Each participant obtained an electronic consent form before the response to the questionnaire.

### Measures

#### Physical Activity

Physical activity frequency was determined by asking about the “physical exercise in the last month when not on vacation.” A detailed operational definition for moderate-to-vigorous physical exercise was included in the questionnaire for better reliability. We grouped these responses as never (0 times), seldom (≤3 times/month), sometimes (4–8 times/month), often (9–20 times/month), and almost every day (≥21 times/month) (Xu et al., 2019).

#### Positive Mental Health

We adopted a widely used single-item happiness measure based on literature (Kye and Park, 2014; Richards et al., 2015). It was adopted from the 36-Item Short-Form Health Survey (SF-36)(Ware and Sherbourne, 1992): “In the past month, how often do you feel happy?” There were five response options recoded into an order-classified variable (1 = never, 2 = rare, 3 = sometimes, 4 = often, 5 = all the time) (Richards et al., 2015).

The job satisfaction of the physicians was measured through the short version of the Minnesota Satisfaction Questionnaire (MSQ) (Weiss et al., 1967; Jiang et al., 2018). The Cronbach's α of MSQ was 0.952.

#### Negative Mental Health

Meanwhile, a Chinese version of the depression anxiety stress scale-21(DASS-21) was used to assess depression, anxiety, and stress symptoms. We used the same cut-off values as reported previously (Jiang et al., 2020). The Cronbach's α of DASS-21 was 0.956.

#### Socio-Demographic and Occupational Characteristics

This part involved socio-demographic and occupational characteristics that were selected based on previous studies (Zhang and Chen, 2019; Felez-Nobrega et al., 2021), including age, gender, marital status, number of children, educational level, professional title, administration position, monthly income, geographical region, outpatient volume/week, night shifts/month, working hours/week, insomnia, and self-assessed health status. Alcohol use and cigarette were also asked.

### Data Analysis

One sample K-S test was used to examine the normality of obtained data. Descriptive analyses about the socio-demographic and occupational characteristics, physical activity, happiness level, job satisfaction, depression, anxiety, and stress status of the sample were conducted.

We used ordinal logistic regression or linear regression analysis to determine the relationship between PA frequency and depression/anxiety/stress/happiness levels or job satisfaction, adjusted by socio-demographic and occupational variables within the whole sample.

All statistical analyses were performed using the STATA software version 16.0 (Stata Corporation, College Station, TX, USA), with the significance level at the *p*-value of 0.05 (two-tailed).

## Results

### Sample Characteristics

In total, 6,986 physicians were invited to participate, 4,576 physicians responded (response rate = 65.5%). Finally, 4,520 completed the questionnaire with no logical errors and were included in the statistical analysis. Their mean age was 38.5 ± 8.6 years, and 41.86% of the physicians were men.

Among them, 1,593 (35.24%) physicians reported having no PA, and 1,772 (39.2%) reported seldom having PA in the previous month. Meanwhile, only 21.88% of physicians reported feeling happy often or always in this sample, and the percentage of participants with normal scores on depression, anxiety, and stress was 55.15, 58.10, and 76.97%, respectively. The MSQ score was 65.89 ± 14.42. Table 1 shows the detailed characteristics.

**Table 1:** Characteristics of physicians in Chinese tertiary psychiatric hospitals (*N* = 4,520).

**Characteristic**	***N***	**%**
**Gender**		
Male	1,892	41.86
Female	2,628	58.14
**Marital status**		
Single	690	15.27
Married	3,668	81.15
Divorced or widowed	162	3.58
**Children**		
None	1,788	39.56
One	2,146	47.48
More than one	586	12.96
**Educational level^a^**		
Bachelor degree or below	2,989	66.13
Master's degree	1,257	27.81
Doctorate degree	274	6.06
**Professional title**		
Junior	1,405	31.08
Middle	1,551	34.31
Senior	1,564	34.60
**Administration position**		
Yes	954	21.11
No	3,566	78.89
**Average monthly income**		
Low (<5,000 RMBs)	720	15.93
Middle (5,000–9,999 RMBs)	2,378	52.61
Upper middle (10,000–20,000 RMBs)	1,308	28.94
High (>20,000 RMBs)	114	2.52
**Region**		
East China	1,860	41.15
Central China	1,463	32.37
West China	1,197	26.48
**Outpatient volume/week**		
0–10	2,033	44.98
11–50	1,254	27.74
>50	1,233	27.28
**Number of charged beds**		
0–10	2,230	49.34
11–20	1,406	31.11
>20	884	19.56
**Night shifts/month**		
0–2	2,022	44.73
3–5	1,718	38.01
>5	780	17.26
**Insomnia**		
No	726	16.06
Seldom (≤3 times/month)	1,369	30.29
Sometimes (1–2 times/week)	1,379	30.51
Often (3–5 times/week)	878	19.42
Daily	168	3.72
**Cigarette use**		
No	3,809	84.27
Former smoker	190	4.20
Current smoker	521	11.53
**Alcohol use**		
Never	2,090	46.24
Sometimes (1–4times/month)	2,256	49.91
Often (>4times/month)	174	3.85
**Health status**		
Very dissatisfied	536	11.86
Dissatisfied	1,628	36.02
Neutral	1,813	40.11
Satisfied	490	10.84
Very satisfied	53	1.17
**Depression**		
Normal	2,493	55.15
Mild	707	15.64
Moderate	1,006	22.26
Serious	156	3.45
Extremely serious	158	3.50
**Anxiety**		
Normal	2,626	58.10
Mild	336	7.43
Moderate	1,046	23.14
Serious	265	5.86
Extremely serious	247	5.46
**Stress**		
Normal	3,479	76.97
Mild	490	10.84
Moderate	303	6.70
Serious	177	3.92
Extremely serious	71	1.57
**PA frequency**		
No	1,593	35.24
Seldom (≤3 times/month)	1,772	39.20
Sometimes (4–8 times/month)	796	17.61
Often (9–20 times/month)	291	6.44
Almost every day (≥21 times/month)	68	1.50
**Happiness**		
Never	121	2.68
Rarely	799	17.68
Sometimes	2,608	57.70
Often	867	19.18
Always	125	2.77
	**Mean**	**SD**
Age (years)	38.50	8.60
Working hours/week	53.01	16.22
Job satisfaction (MSQ)	65.89	14.42

a *In China, medical school graduates are awarded a bachelor's degree in medicine (similar to the European and Russian systems). Some obtained a master's or doctorate degree in addition to their medical degree*.

### Association of PA Frequency and Mental Health

In the ordinal logistic regression, PA frequency (seldom/often, reference: No PA) was significantly associated with depression (PA sometimes as well) and anxiety, whereas PA almost every day was not significantly associated with them (Table 2).

**Table 2:** Ordinal logistic regression examining factors associated with depression, anxiety, and stress (*N* = 4,520).

	**Depression**	**Anxiety**	**Stress**
	**OR (95%CI)**	**OR (95%CI)**	**OR (95%CI)**
**PA frequency (ref. No)**			
Seldom	0.74 (0.65–0.85)^**^	0.85 (0.74–0.98)^*^	0.85 (0.72–1.01)
Sometimes	0.75 (0.62–0.90)^*^	0.83 (0.69–1.01)	0.79 (0.63–1.00)
Often	0.51 (0.38–0.70)^**^	0.70 (0.52–0.94)^*^	0.82 (0.56–1.20)
Almost every day	1.00 (0.57–1.77)	0.66 (0.34–1.28)	0.80 (0.36–1.75)
**Gender (ref. Male)**	0.92 (0.79–1.08)	0.97 (0.83–1.14)	0.96 (0.80–1.16)
**Marital status (ref. Single)**			
Married	0.81 (0.64–1.02)	0.91 (0.73–1.15)	1.05 (0.79–1.38)
Divorced or widowed	1.16 (0.80–1.68)	0.93 (0.63–1.36)	1.23 (0.78–1.94)
Children (ref. No)			
One	0.98 (0.84–1.16)	0.93 (0.79–1.10)	0.88 (0.72–1.07)
More than one	0.95 (0.76–1.18)	0.87 (0.69–1.09)	0.78 (0.59–1.02)
**Educational level (ref. Bachelor degree or below)**			
Master's degree	0.88 (0.76–1.02)	0.85 (0.73–0.99)^*^	1.00 (0.84–1.20)
Doctorate degree	0.64 (0.48–0.86)^*^	0.70 (0.52–0.95)^*^	0.56 (0.37–0.84)^**^
**Professional title (ref. Junior)**			
Middle	1.21 (1.00–1.45)^*^	1.08 (0.90–1.30)	1.14 (0.92–1.42)
Senior	1.36 (1.06–1.74)^*^	1.22 (0.95–1.58)	1.17 (0.86–1.59)
**Administration position (ref. No)**	0.65 (0.54–0.79)^**^	0.78 (0.64–0.95)^*^	0.99 (0.78–1.26)
**Average monthly income (ref. Low)**			
Middle	0.78 (0.65–0.93)^**^	0.77 (0.65–0.92)^**^	0.90 (0.72–1.11)
Upper middle	0.81 (0.65–1.00)	0.64 (0.52–0.80)^**^	0.78 (0.60–1.02)
High	0.89 (0.58–1.38)	0.85 (0.55–1.32)	0.99 (0.57–1.73)
**Region (ref. East China)**			
Central China	1.10 (0.95–1.28)	1.16 (0.99–1.35)	1.08 (0.90–1.30)
West China	1.25 (1.06–1.47)^**^	1.07 (0.91–1.26)	1.11 (0.91–1.36)
**Outpatient volume/week (ref. 0–10)**			
11–50	1.00 (0.86–1.17)	0.94 (0.80–1.10)	0.81 (0.67–0.99)^*^
>50	0.94 (0.80–1.11)	0.93 (0.79–1.10)	0.87 (0.71–1.07)
**Number of charged beds (ref. 0–10)**			
11–20	1.13 (0.97–1.31)	0.99 (0.85–1.15)	1.01 (0.85–1.21)
>20	0.97 (0.82–1.14)	0.93 (0.78–1.10)	0.89 (0.72–1.09)
**Night shifts/month (ref. 0–2)**			
3–5	1.09 (0.94–1.26)	1.04 (0.89–1.21)	1.14 (0.95–1.37)
>5	1.11 (0.92–1.33)	1.05 (0.87–1.26)	1.11 (0.89–1.38)
**Insomnia (ref. No)**			
Seldom	2.13 (1.71–2.64)^**^	2.50 (1.99–3.14)^**^	2.14 (1.57–2.92)^**^
Sometimes	1.58 (1.27–1.96)^**^	1.74 (1.38–2.18)^**^	1.57 (1.14–2.15)^**^
Often	3.65 (2.89–4.60)^**^	4.25 (3.33–5.42)^**^	4.24 (3.08–5.83)^**^
Daily	5.09 (3.55–7.29)^**^	6.94 (4.83–9.97)^**^	7.72 (5.10–11.69)^**^
**Cigarette use (ref. No)**			
Used but quit	1.40 (1.03–1.89)^*^	1.37 (1.00–1.87)^*^	1.12 (0.78–1.61)
Yes	1.28 (1.04–1.57)^*^	1.23 (0.99–1.52)	1.02 (0.79–1.32)
**Alcohol use (ref. Never)**			
Sometimes	1.36 (1.18–1.56)^**^	1.16 (1.01–1.34)^*^	1.13 (0.95–1.34)
Often	1.81 (1.30–2.54)^**^	1.61 (1.15–2.25)^**^	1.98 (1.36–2.89)^**^
**Health status (ref. Very dissatisfied)**			
Dissatisfied	0.46 (0.38–0.55)^**^	0.44 (0.36–0.53)^**^	0.46 (0.38–0.57)^**^
Neutral	0.24 (0.20–0.30)^**^	0.22 (0.18–0.27)^**^	0.24 (0.19–0.30)^**^
Satisfied	0.09 (0.06–0.12)^**^	0.08 (0.06–0.11)^**^	0.09 (0.06–0.15)^**^
Very satisfied	0.03 (0.01–0.11)^**^	0.05 (0.01–0.16)^**^	0.04 (0.01–0.32)^**^
**Age (years)**	1.00 (0.99–1.01)	0.99 (0.98–1.01)	0.98 (0.97–1.00)^*^
**Working hours/week**	1.00 (1.00–1.01)	1.01 (1.00–1.01)^**^	1.01 (1.00–1.01)^**^

* *p < 0.05*,

** *p < 0.001*.

Physical activity frequency was also not associated with job satisfaction of physicians (Table 3).

**Table 3:** Regression examining factors associated with job satisfaction (*N* = 4,520).

**Subjective happiness**	**Coefficient**	**95.0% CI (Lower)**	**95.0% CI (Upper)**	***p***
**PA frequency (ref. No)**				
Seldom	0.48	−0.46	1.41	0.316
Sometimes	1.05	−0.16	2.26	0.090
Often	1.71	−0.06	3.49	0.058
Almost every day	−1.10	−4.49	2.29	0.525
**Gender (ref. Male)**	0.97	−0.04	1.98	0.060
**Marital status (ref. Single)**				
Married	1.42	−0.09	2.92	0.065
Divorced or widowed	−0.80	−3.34	1.74	0.537
**Children (ref. No)**				
One	−0.84	−1.90	0.22	0.121
More than one	−1.02	−2.48	0.44	0.170
**Educational level (ref. Bachelor degree or below)**				
Master's degree	−0.01	−0.98	0.96	0.983
Doctorate degree	−0.48	−2.27	1.31	0.600
**Professional title (ref. Junior)**				
Middle	−2.32	−3.52	−1.11	**<0.001**
Senior	−2.81	−4.46	−1.16	**0.001**
**Administration position (ref. No)**	4.65	3.41	5.88	**<0.001**
**Average monthly income (ref. Low)**				
Middle	0.22	−0.96	1.41	0.712
Upper middle	2.14	0.70	3.58	**0.004**
High	3.75	0.94	6.55	**0.009**
**Region (ref. East China)**				
Central China	−0.63	−1.61	0.35	0.208
West China	−0.86	−1.93	0.21	0.116
**Outpatient volume/week (ref. 0–10)**				
11–50	0.13	−0.92	1.17	0.814
>50	0.64	−0.45	1.73	0.251
**Number of charged beds (ref. 0–10)**				
11–20	−0.62	−1.60	0.36	0.216
>20	0.00	−1.11	1.10	0.998
**Night shifts/month (ref. 0–2)**				
3–5	0.31	−0.66	1.27	0.536
>5	−1.61	−2.86	−0.37	**0.011**
**Insomnia (ref. No)**				
Seldom	−2.20	−3.48	−0.92	**0.001**
Sometimes	−1.33	−2.57	−0.09	**0.035**
Often	−4.11	−5.55	−2.67	**<0.001**
Daily	−6.50	−8.89	−4.11	**<0.001**
**Cigarette use (ref. No)**				
Used but quit	0.23	−1.84	2.30	0.825
Yes	−1.68	−3.08	−0.27	**0.020**
**Alcohol use (ref. Never)**				
Sometimes	−1.23	−2.15	−0.32	**0.008**
Often	−2.86	−5.10	−0.63	**0.012**
**Health status (ref. Very dissatisfied)**				
Dissatisfied	3.68	2.31	5.04	**<0.001**
Neutral	7.08	5.68	8.48	**<0.001**
Satisfied	10.97	9.16	12.78	**<0.001**
Very satisfied	16.36	12.41	20.31	**<0.001**
Age (years)	−0.09	−0.17	−0.01	**0.037**
Working hours/week	0.01	−0.01	0.04	0.318

*Bold value for p < 0.05*.

Physicians who had more frequent PA were more likely to report feeling happy, while PA almost every day was not associated with happiness (Table 4).

**Table 4:** Ordinal logistic regression examining factors associated with subjective happiness (*N* = 4,520).

**Subjective happiness**	**OR**	**95.0% CI (Lower)**	**95.0% CI (Upper)**	***p***
**PA frequency (ref. No)**				
Seldom	1.25	1.08	1.43	**0.002**
Sometimes	1.39	1.16	1.67	**0.000**
Often	1.83	1.40	2.39	**0.000**
Almost every day	1.30	0.78	2.17	0.310
**Gender (ref. Male)**	1.44	1.24	1.68	**<0.001**
**Marital status (ref. Single)**				
Married	1.39	1.10	1.74	**0.005**
Divorced or widowed	0.70	0.48	1.02	0.067
**Children (ref. No)**				
One	0.90	0.77	1.06	0.198
More than one	1.10	0.88	1.36	0.413
**Educational level (ref. Bachelor degree or below)**				
Master's degree	1.02	0.88	1.18	0.763
Doctorate degree	1.01	0.77	1.32	0.969
**Professional title (ref. Junior)**				
Middle	0.95	0.79	1.13	0.547
Senior	0.90	0.70	1.15	0.385
**Administration position (ref. No)**	1.83	1.52	2.21	**<0.001**
**Average monthly income (ref. Low)**				
Middle	0.98	0.82	1.17	0.814
Upper middle	1.00	0.81	1.24	0.995
High	1.08	0.71	1.65	0.720
**Region (ref. East China)**				
Central China	0.96	0.82	1.11	0.542
West China	0.88	0.75	1.03	0.114
**Outpatient volume/week (ref. 0–10)**				
11–50	0.89	0.76	1.04	0.148
>50	0.93	0.79	1.10	0.419
**Number of charged beds (ref. 0–10)**				
11–20	0.94	0.81	1.09	0.384
>20	0.92	0.78	1.09	0.355
**Night shifts/month (ref. 0–2)**				
3–5	1.04	0.90	1.20	0.600
>5	0.91	0.75	1.09	0.308
**Insomnia (ref. No)**				
Seldom	0.56	0.46	0.68	**<0.001**
Sometimes	0.83	0.68	1.00	**0.047**
Often	0.34	0.27	0.43	**<0.001**
Daily	0.19	0.13	0.27	**<0.001**
**Cigarette use (ref. No)**				
Used but quit	0.84	0.61	1.15	0.276
Yes	1.06	0.86	1.31	0.592
**Alcohol use (ref. Never)**				
Sometimes	0.84	0.73	0.96	**0.010**
Often	0.99	0.71	1.40	0.974
**Health status (ref. Very dissatisfied)**				
Dissatisfied	2.35	1.92	2.89	**<0.001**
Neutral	4.77	3.83	5.93	**<0.001**
Satisfied	23.32	17.56	30.99	**<0.001**
Very satisfied	168.78	89.32	318.94	**<0.001**
**Age (years)**	0.99	0.98	1.00	0.195
Working hours/week	1.00	0.99	1.00	0.116

*Bold value for p < 0.05*.

Further analysis showed that those who reported PA almost every day (*N* = 68) had the following features compared to those who did not report PA nearly every day: they were predominantly men (64.71 vs. 41.51%, *p* < 0.001), older (47.94 ± 10.58 vs. 38.36 ± 8.48 years, *p* < 0.001), and had significantly more smokers (16.18 vs. 11.46% current smokers, 11.76 vs. 4.09% past smokers, *p* < 0.05) and frequent alcohol users (>4 times/month) (10.29 vs. 3.75%, *p* < 0.05).

## Discussion

To our best knowledge, this study was one of the first to focus on the relationship between PA and mental health among Chinese physicians. This research demonstrated that PA frequency was positively associated with depression, anxiety, and happiness after controlling for relevant confounders, aligned with some other studies (Fisher et al., 2019; Kroencke et al., 2019; Xu et al., 2019; Zhang and Chen, 2019; Stevens et al., 2021; Ye et al., 2021).

One intriguing finding in this study is that the most frequent PA group (almost every day) was not significantly associated with depression, anxiety, or happiness. This may sound counterintuitive, but there are several possible explanations. First, individuals who reported highly frequent PA may represent a unique group, which might resemble the so-called “compulsive exercise” (Hausenblas and Downs, 2002). Second, most people, especially those as busy as physicians, do not have time to have PA almost every day. Highly frequent PA would consume much time and may affect the work–life balance (Gragnano et al., 2020), which may in turn decrease the level of mental health (Wan Mohd Yunus et al., 2020). This interesting finding needs further investigation (Zhang and Chen, 2019).

Physical activity might improve mental health through several physiological or psychological pathways (Zhang and Chen, 2019). For example, a previous study demonstrated that mobility and cognition explained the largest proportion of the association (Felez-Nobrega et al., 2021). According to a national survey, Chinese physicians have appropriate mobility and cognition in their daily life (Wu et al., 2019).

The overall level of mental health in Chinese physicians in this study was lower than samples from other reports. For example, the prevalence of generalized anxiety among American physicians was 14.9 and 11.7% for depression, much lower than our results (Sonis et al., 2021). Participants from 15 European Union countries reported 82.9% of them being happy (all the time, very often, or often) in the past month (Richards et al., 2015), against 21.88% in our research. Another research in Chinese residents demonstrated that 71.2% of participants had high subjective well-being or happiness (Xu et al., 2019). Given the value of regular PA to mental health, Chinese physicians should lead the way in adopting PA to get better mental health (Yancey et al., 2013).

This research has several limitations. First, as it was a crosssectional survey, causal relations cannot be inferred. Second, the samples in this study were from tertiary psychiatric hospitals, and so the results may not be generalized to all Chinese physicians. Third, happiness and PA frequency in this study were assessed using two single-item self-reported questions. Some potentially important information, such as the type and duration of PA, was not included. The reliability and validity of the evaluation may also be limited. Fourth, as inherent to this type of study, recall and response bias cannot be ruled out.

## Conclusions

The current study showed the level of mental health in Chinese physicians in psychiatric hospitals is low. We also found an independent association of higher PA frequency (except PA almost every day) with depression/anxiety and happiness. Increasing PA frequency may promote mental health in Chinese physicians in tertiary psychiatric hospitals. Policy makers and hospital management should focus on interventions, including programs aiming to promote PA and mental health.

## Data Availability Statement

The raw data supporting the conclusions of this article will be made available by the authors, without undue reservation.

## Ethics Statement

The studies involving human participants were reviewed and approved by the Ethics Committee in Chaohu Hospital of Anhui Medical University. The patients/participants provided their written informed consent to participate in this study. Written informed consent was obtained from the individual(s) for the publication of any potentially identifiable images or data included in this article.

## Author Contributions

HL, FJ, and Y-LT made substantial contributions to the study design. YL collected data. JL analyzed the data. JL and FJ interpreted the results of analysis and completed the manuscripts. All authors have read and approved the published version of the manuscript.

## Funding

This research was funded by the Beijing Medical and Health Foundation (Grant No. MH180924).

## Conflict of Interest

The authors declare that the research was conducted in the absence of any commercial or financial relationships that could be construed as a potential conflict of interest.

## Publisher's Note

All claims expressed in this article are solely those of the authors and do not necessarily represent those of their affiliated organizations, or those of the publisher, the editors and the reviewers. Any product that may be evaluated in this article, or claim that may be made by its manufacturer, is not guaranteed or endorsed by the publisher.
